# Analytical thermal model for end-pumped solid-state lasers

**DOI:** 10.1007/s00340-017-6848-y

**Published:** 2017-11-04

**Authors:** L. Cini, J. I. Mackenzie

**Affiliations:** 1 Dipartimento di Fisica, Università di Pisa, Largo B. Pontecorvo 3, Pisa, 56127 Italy; 2 Optoelectronics Research Centre, University of Southampton, Highfield Southampton SO17 1BJ, Southampton, United Kingdom

## Abstract

Fundamentally power-limited by thermal effects, the design challenge for end-pumped “bulk” solid-state lasers depends upon knowledge of the temperature gradients within the gain medium. We have developed analytical expressions that can be used to model the temperature distribution and thermal-lens power in end-pumped solid-state lasers. Enabled by the inclusion of a temperature-dependent thermal conductivity, applicable from cryogenic to elevated temperatures, typical pumping distributions are explored and the results compared with accepted models. Key insights are gained through these analytical expressions, such as the dependence of the peak temperature rise in function of the boundary thermal conductance to the heat sink. Our generalized expressions provide simple and time-efficient tools for parametric optimization of the heat distribution in the gain medium based upon the material and pumping constraints.

## Introduction

The end-pumped solid-state laser is a mature design architecture exploited for many scientific, industrial, and medical laser applications, in the tens-of-watts power regime. Cost effective, compact, and relatively efficient, in recent decades their performance has capitalized on improving diode-laser brightness and power. Further power-scaling, however, is fundamentally limited by the thermo-optical properties of the gain medium and the induced optical distortions, primarily driven by the quantum defect between pump and the emission wavelengths. Alternatively, the geometry of the gain medium can be optimized to enhance thermal management and minimize the thermal-lensing effects, such as the thin-disk [1], fibre [2], or slab architectures [3], which have demonstrated multi-kW average powers. Nonetheless, for many applications, the end-pumped bulk architecture still holds an important position for its simplicity and robust performance.

The basic design strategy for these lasers has been to try to mitigate the effects of the induced thermal-lensing and aberrations [4]. As such, it is important to understand the induced temperature distribution over the active volume of interest, that is, where the cavity mode passes through the excited region of the gain medium. Typically, this involves numerical simulations solving the heat equation with finite-element algorithms, having almost completely replaced analytical solutions due to the complexity of the necessary assumptions made to obtain exact expressions. Analytical solutions, though, are unquestionably important: they highlight qualitative and quantitative features of underlying physical phenomena and provide more accurate solutions in far less time than numerical calculations, particularly if trying to perform parameter-dependence studies. One strict assumption generally made in determining the exact solution for the temperature profile along an end-pumped solid-state laser is that the thermal conductivity of the crystal is not significantly dependent on temperature [5]. While a reasonable assumption for many active media, operating at and above room temperature (RT), it is generally not valid when cooled to a cryogenic temperature (CT) [6]. Here, the gradient for the changing thermal conductivity with respect to temperature steepens considerably compared with the same around RT. With this argument in mind, and, therefore, including a temperature dependence for the thermal conductivity in the heat conduction equation, it is possible to exploit integral transforms to reach analytical solutions [7].

In this paper, we use the Kirchhoff transform [8] to convert the non-linear heat equation into a solvable linear equation for a cylindrical radially isotropic gain element. Analytical solutions for the temperature distribution along the length of a side-cooled end-pumped rod are presented for different pump distributions that can be used for practical configurations, such as near-diffraction-limited, to fibre-coupled diode-laser, pumps. Furthermore, this result provides novel analytical expressions for the thermal-lens strength associated with the pump-induced accumulated optical phase shift, which converge to well-known equations [5] when a temperature-independent thermal conductivity is chosen.

The rest of this paper is organized as follows: we start by introducing the model for the thermal conductivity *k*(*T*), which matches with actual measurements and provides simple solutions for the Kirchhoff transform, and its dependence over the two main temperature ranges of practical interest. Utilizing this form for *k*(*T*), the derivation of the exact solutions for the temperature distribution along an end-pumped rod is given. Four different pump distributions are studied to cover commonly used pump sources, and to make direct comparison with previous work; these include the top-hat, Gaussian, generalized *n*th order super-Gaussian, and annular (donut) distributions. At this point, the importance of the heat transfer coefficient, *h*, at the boundary between rod and heat sink, is highlighted. An expression is derived that relates the pumping parameters to a minimum critical value for *h*, below which, the temperature at the center of rod rises rapidly. In the penultimate section, the expressions for the thermal-lens strength, now calculable at any temperature in the CT and well above RT regimes, are derived, and then compared with the two extremes reported in the literature, i.e., top-hat and Gaussian. The solution for the Gaussian pump is derived as a special solution to the generalized SG distribution. Finally, to end the paper, we summarize with conclusions and appendices for detailed workings.

## Temperature dependence of thermal conductivity

**Fig. 1:**
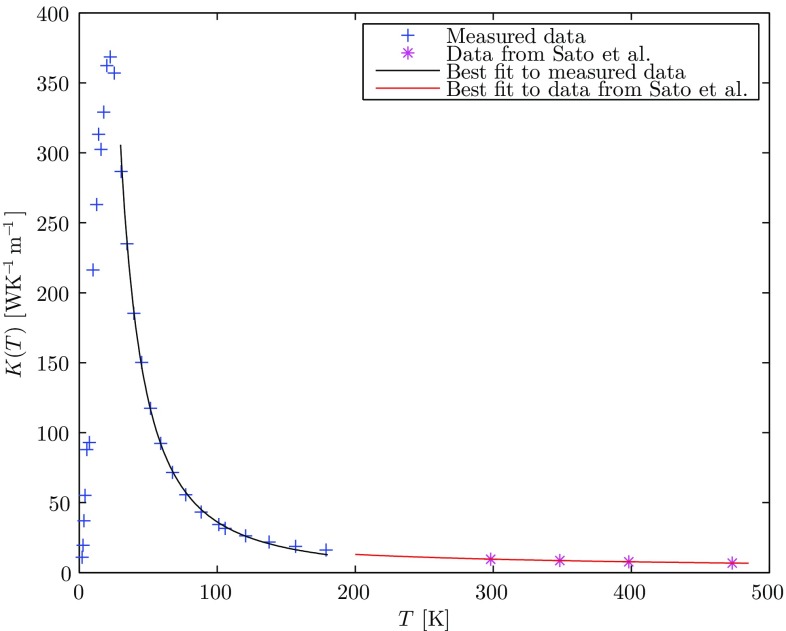
Measured thermal conductivity dependence on temperature, after [10, 11], and best fits using Eq. (1)

We focus our attention on the Nd:YAG crystal. Since YAG is a cubic crystal, the thermal conductivity can be considered as a scalar quantity. The dependence on temperature of the thermal conductivity can be modeled by the following formula:

1 $$\begin{aligned} k(T)=k_0\left( \frac{T}{T_0}\right) ^m, \end{aligned}$$

where $k_0$, $T_0$ and *m* result from a best fit to measured data. Since these parameters strictly depend on the doping level of the rare-earth ion, we choose a particular doping concentration, say ${\sim }1\%$. We performed two best fits for two different temperature ranges around CT and RT. The first fit is based on measured data on a 1.3 at.% Nd:YAG sample in a temperature range of 40 K$\le T\le$175 K [10], while the second is based on published data of Sato et al. [11] relating to 1.2 at.% Nd:YAG in a temperature range of 300 K$\le T\le$475 K. By finding the intersection of the two fitting curves, a common value of $T_0$ and $k_0=k(T_0)$ for the two temperature ranges can be found. The values of the parameters for ${\sim }$1 at.% Nd:YAG are, therefore, as follows: $T_0=164.17$ K, $k_0=15.09$ WK$^{-1}$ m$^{-1}$, $m_{\text {CT}}=-1.77$, and $m_{\text {RT}}=-0.75$. The data used and the corresponding best fits curves are shown in Fig. 1.

### Temperature dependence of thermal conductivity for selected materials

In Table 1, the values of $k_0$, $T_0$, and *m* are provided for Nd:YAG at different temperatures, along with different doping concentrations and a variety of other interesting host materials, including some that would not normally be considered for an end-pumped rod architecture, rather more appropriate to a thin-disk geometry. These values result from a best fit of Eq. (1) to available data from the published literature [6, 11, 12]. As previously mentioned, the thermal conductivity can be considered a scalar quantity strictly for isotropic laser crystals. However, since, in this thermal model, a pure radial heat flux will be assumed (see Sect. 3), uniaxial laser materials can also be included and, in this case, only the dependence on temperature along the a-axes is considered.

**Table 1:** Thermal conductivity parameters for selected materials

Crystal	*T* [K]	$k_0$ $\left[ \frac{W}{Km}\right]$	$T_0$ [K]	*m*
YAG$^\mathrm{a}$	80–300	17.29	199.96	−1.57
1.3 at.% Nd:YAG	40–175	15.09	164.17	−1.77
2 at.% Yb:YAG$^\mathrm{a}$	80–300	12.59	199.45	−1.46
4 at.% Yb:YAG$^\mathrm{a}$		11.72	199.38	−1.17
15 at.% Yb:YAG$^\mathrm{a}$		9.08	199.25	−0.87
LuAG$^\mathrm{a}$		12.06	199.43	−1.14
YLF(a)$^\mathrm{a}$		8.31	199.17	−1.57
5 at.% Yb:YLF(a)$^\mathrm{a}$		5.74	199.24	−1.04
LuLF(a)$^\mathrm{a}$		7.86	199.13	−1.65
GGG$^\mathrm{b}$	100–300	12.19	199.44	−1.25
CY$_2$O$_3$ $^\mathrm{b}$		20.43	200.37	−1.19
GdVO$_4$(a)$^\mathrm{b}$		13.71	199.57	−1.30
YAG$^\mathrm{c}$	300–475	14.00	199.58	−0.78
0.7 at.% Nd:YAG$^\mathrm{c}$		13.55	199.56	−0.77
0.9 at.% Nd:YAG$^\mathrm{c}$		13.36	199.56	−0.77
1.2 at.% Nd:YAG$^\mathrm{c}$		15.09	164.17	−0.75
5.0 at.% Yb:YAG$^\mathrm{c}$		8.98	199.22	−0.56
9.4 at.% Yb:YAG$^\mathrm{c}$		8.12	199.19	−0.54
22.9 at.% Yb:YAG$^\mathrm{c}$		6.86	199.16	−0.48

References: $$^{\mathrm{a}}$$ [6], $$^{\mathrm{b}}$$ [12], and $$^{\mathrm{c}}$$ [11]

## Analytical solution

**Fig. 2:**
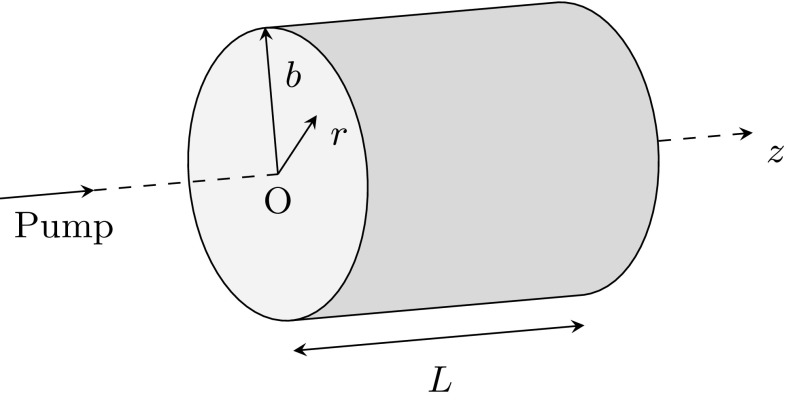
End-pumped laser rod representation. Point O is located at $r=0$ and $z=0$

The steady-state heat conductance equation, with temperature-dependent thermal conductivity and different end-pumping profiles, governing the system shown in Fig. 2, is:

2 $$\begin{aligned} \mathbf {\nabla }\cdot \left[ k(T)\mathbf {\nabla }T(r,z) \right] +S(r,z)=0, \end{aligned}$$

where *k*(*T*) is the temperature-dependent thermal conductivity and *S*(*r*, *z*) is the thermal power per unit volume dissipated in the laser rod.

The edge of the rod is considered to be surrounded by a heat sink, held at a constant temperature, $T_c$, by active cooling. Heat transfer across the boundary between the laser rod and the heat sink is described by the heat transfer coefficient or surface conductance, *h*. The end faces are instead supposed to be in contact with air, with negligible heat flux through them. An equivalent $h_{\text {ef}}$ coefficient can be calculated for the end faces [15], and since typically $h>>h_{\text {ef}}$, the assumption of pure radial heat flux can be made [5]. Furthermore, it is assumed in the following that the diameter of the pumped region is significantly smaller than the inverse of the absorption coefficient, reinforcing the statement of radial heat flux. It follows that the longitudinal derivatives in Eq. (2) with respect to the corresponding radial derivatives [14] can be neglected. Here, it is also assumed that the pump profile is axisymmetric, the behavior of the thermal conductivity *k*(*T*) of the crystal is described by Eq. (1), and the cooling is isotropic in the *z*-plane. These assumptions allow us to write Eq. (2) as follows:

3 $$\begin{aligned} \frac{1}{r}\frac{\mathrm{d}}{\mathrm{d}r}\left[ rk(T)\frac{\mathrm{d}T(r,z)}{\mathrm{d}r}\right] +S(r,z)=0. \end{aligned}$$

Equation (3) can be solved introducing an integral transform (Kirchhoff transform) in terms of a function *U* defined as follows [8]:

4 $$\begin{aligned} U(r,z)=\int ^{T}k(\tau )\mathrm{d}\tau . \end{aligned}$$

By the fundamental theorem of calculus,

5 $$\begin{aligned} k(T)=\frac{\mathrm{d}U}{\mathrm{d}T} \end{aligned}$$

and, substituting Eq. (5) into Eq. (3) and using the chain rule of differentiation, Eq. (3) is transformed into the linear equation:

6 $$\begin{aligned} \nabla ^2U(r,z)=\frac{1}{r}\frac{\mathrm{d}U(r,z)}{\mathrm{d}r}+\frac{\mathrm{d}^2U(r,z)}{\mathrm{d}r^2}=-S(r,z) \end{aligned}$$

that can be easily solved for different pump distributions and the actual temperature can be determined by the inverse Kirchhoff transform.

Using Eq. (1), one obtains

7 $$\begin{aligned} U=\frac{k_0}{(m+1)T_0^m}T^{m+1}+C, \end{aligned}$$

where *C* is an arbitrary constant of integration. It is important to note that Eq. (7) is not valid if $m=-1$; however, the temperature distribution in this case has already been derived by Hodgson and Weber for a side-pumped laser rod [9].

**Fig. 3:**
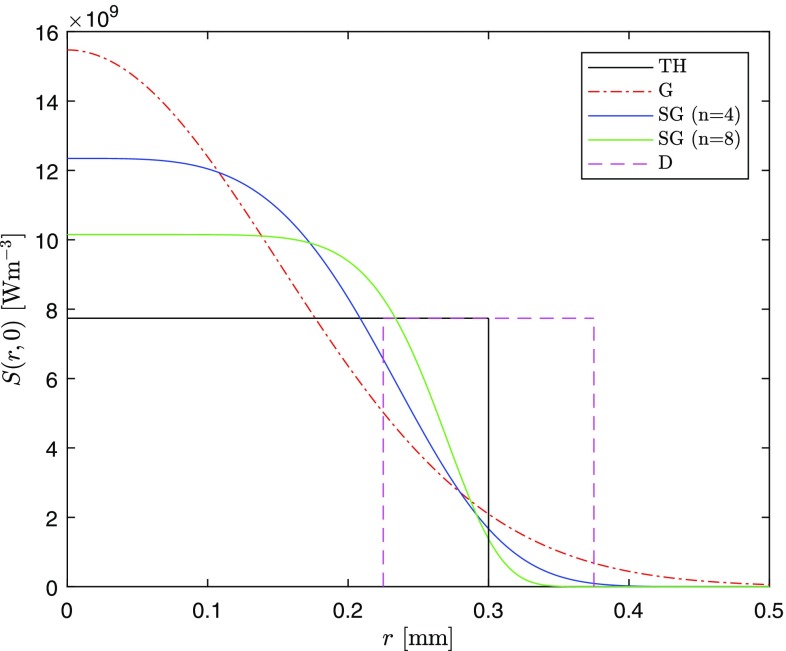
Different heat source distributions considered. The total heating power for each is 5.16 W

The four different pump distributions studied in this paper, corresponding to four different *S*(*r*, *z*) terms in Eq. (6), are shown in Fig. 3 at $z=0$ (see Sect. 3.1 for the value of the parameters considered). They are, respectively, the top-hat (TH), Gaussian (G), super-Gaussian (SG), and donut (D). Two super-Gaussian profiles are reported in the figure, one for the fourth order and the other for the eighth order.

### Top-hat pumping

Although not a typically realistic pump distribution for end-pumped lasers, the top-hat profile is the simplest starting solution, which can be easily compared with the previous literature studies [5, 15]. Consider the following thermal loading distribution:

8 $$\begin{aligned} S(r,z)={\left\{ \begin{array}{ll} Q_0e^{-\alpha z} &{} \quad 0\le r\le a \\ 0 &{} \quad a< r\le b, \end{array}\right. } \end{aligned}$$

where $Q_0$ is a normalization constant, $\alpha$ is the pump absorption coefficient, *a* is the radius of the pumping beam, and *b* is the radius of the laser rod. The normalization constant $Q_0$ can be calculated using the following relation:

9 $$\begin{aligned} Q_0=\frac{\eta _h P}{V}, \end{aligned}$$

where $\eta _h P$ is the total heating power ($\eta _h$ is the fractional thermal load) and *V* is the volume of the pump-photon distribution in the rod, where it is assumed that there are insignificant energy migration effects and the heat source is created at the point of pump-photon absorption, that is

10 $$\begin{aligned} V=\int _0^{2\pi }\mathrm{d}\varphi \int _0^a r\mathrm{d}r \int _0^Le^{-\alpha z}\mathrm{d}z=\frac{\pi a^2 \eta _{\mathrm{abs}}}{\alpha }, \end{aligned}$$

where $1-e^{-\alpha L}=\eta _{\mathrm{abs}}$ is the absorption efficiency. Substituting Eqs. (9) and (10) into Eq. (8), the thermal power per unit volume dissipated into the laser rod becomes:

11 $$\begin{aligned} S(r,z)={\left\{ \begin{array}{ll} \frac{\eta _h P_{\text {in}}\alpha e^{-\alpha z}}{\pi a^2} &{} \quad 0\le r\le a \\ 0 &{} \quad a< r\le b, \end{array}\right. } \end{aligned}$$

where $P_{\text {in}}$ is the incident pump power.

Equation (6) is solved separately between $0\le r\le a$ and $a<r\le b$, resulting in two functions $U_1$ and $U_2$, respectively, leading to two temperature solutions, $T_1(r,z)$ and $T_2(r,z)$ in the respective regions, as shown in Appendix A. These solutions are as follows:

12 $$\begin{aligned} T_1(r,z)=\left\{ F_0e^{-\alpha z}\left[ 1-\frac{r^2}{a^2}+\ln \left( \frac{b^2}{a^2} \right) \right] +\left( T_c+\frac{\eta _h P_{\text {in}} \alpha e^{-\alpha z}}{2\pi bh} \right) ^{m+1} \right\} ^{\frac{1}{m+1}} \end{aligned}$$

for $$0\le r\le a$$ and

13 $$\begin{aligned} T_2(r,z)=\left[ F_0e^{-\alpha z}\ln \left( \frac{b^2}{r^2}\right) + \left( T_c+\frac{\eta _h P_{\text {in}} \alpha e^{-\alpha z}}{2\pi bh}\right) ^{m+1} \right] ^{\frac{1}{m+1}} \end{aligned}$$

for $a<r\le b$, where $F_0$ is a constant defined as follows:

14 $$\begin{aligned} F_0=\frac{\eta _h P_{\text {in}} \alpha (m+1)T_0^m}{4 \pi k_0}. \end{aligned}$$

**Fig. 4:**
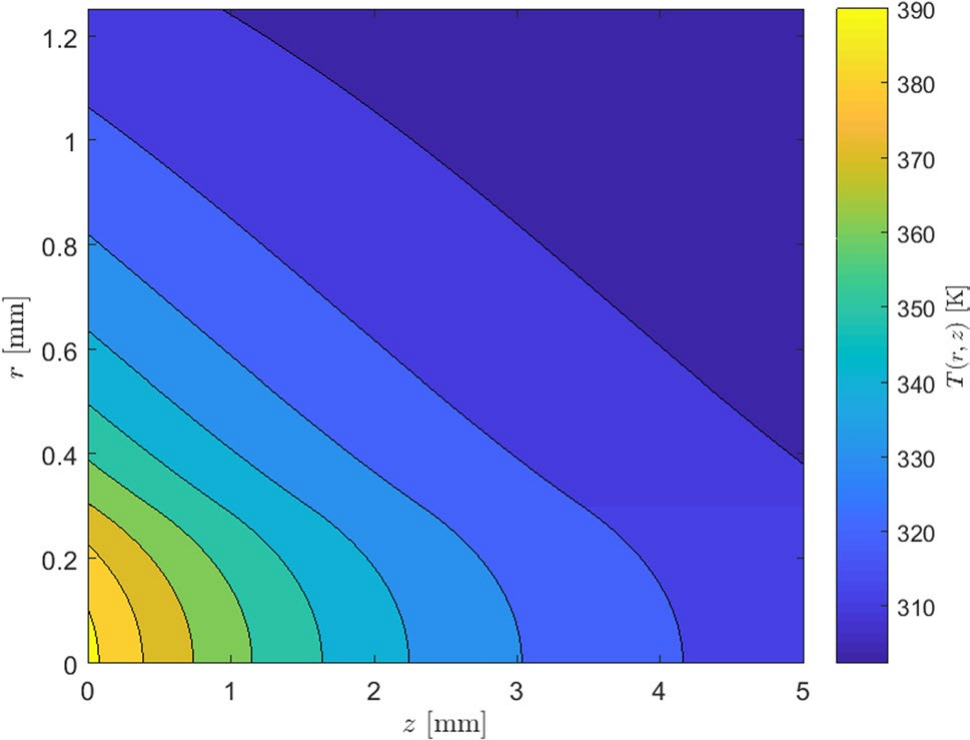
Temperature profile of a top-hat end-pumped laser rod

Figure 4 shows the calculated temperature distribution using Eqs. (12) and (13) with the following parameters: $b=1.25$, $L=5$ mm, $a=300$ $\upmu$m, $P_{\text {in}}=25$ W, $\eta _h=0.25$, $T_c=300$ K, $\alpha =350$ m$^{-1}$ and $h=2$ WK$^{-1}$cm$^{-2}$ (the choice of this value for *h* will be discussed in Sect. 4).

**Fig. 5:**
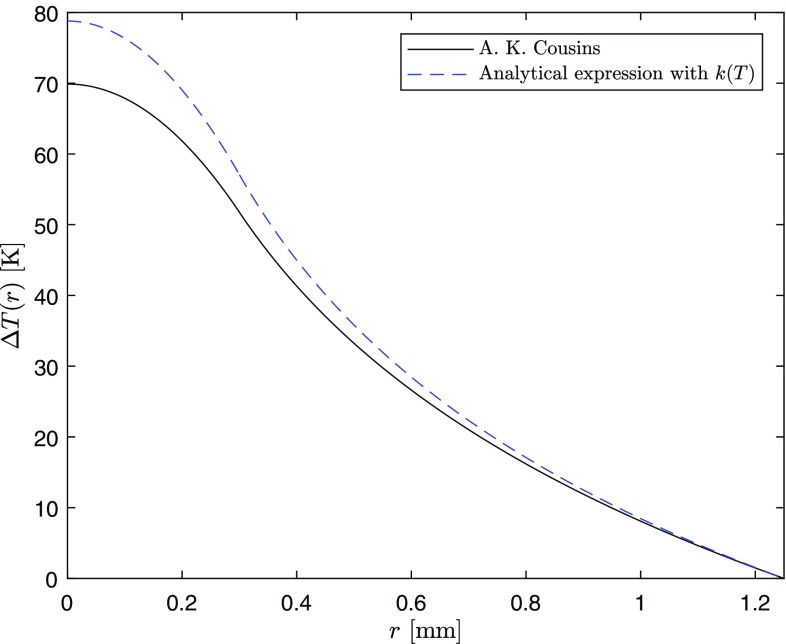
Comparison between our analytical temperature difference profile in the case of RT top-hat pumping and the one published by Cousins [15]

The temperature-independent thermal conductivity case can be obtained from Eqs. (12) and (13) setting $m=0$. In this way, the temperature distribution inside the rod is given by the following:

15 $$\begin{aligned} \Delta T(r,z)=\frac{\eta _h P_{\text {in}} \alpha e^{-\alpha z}}{4 \pi k_0}{\left\{ \begin{array}{ll} {1-\frac{r^2}{a^2}+\ln \left( \frac{b^2}{a^2}\right) } &{} \quad 0\le r\le a \\ {\ln \left( \frac{b^2}{r^2}\right) } &{} \quad a< r\le b, \end{array}\right. } \end{aligned}$$

where $\Delta T(r,z)=T(r,z)-T(b,z)$. Equation (15) shows the well-known quadratic radial dependence of the temperature inside the pumped region of the rod and the logarithmic dependence outside [15]. In Fig. 5, a comparison, at the pumped surface of the rod, $z=0$, for $\Delta T(r,z)$ considering a temperature-dependent thermal conductivity, and Eq. (15), is shown. The parameters for this comparison are the same as those for Fig. 4. The constant thermal conductivity in Eq. (15) is evaluated using Eq. (1) at $T=T_c$.

#### Temperature profile for different coolant temperatures

**Fig. 6:**
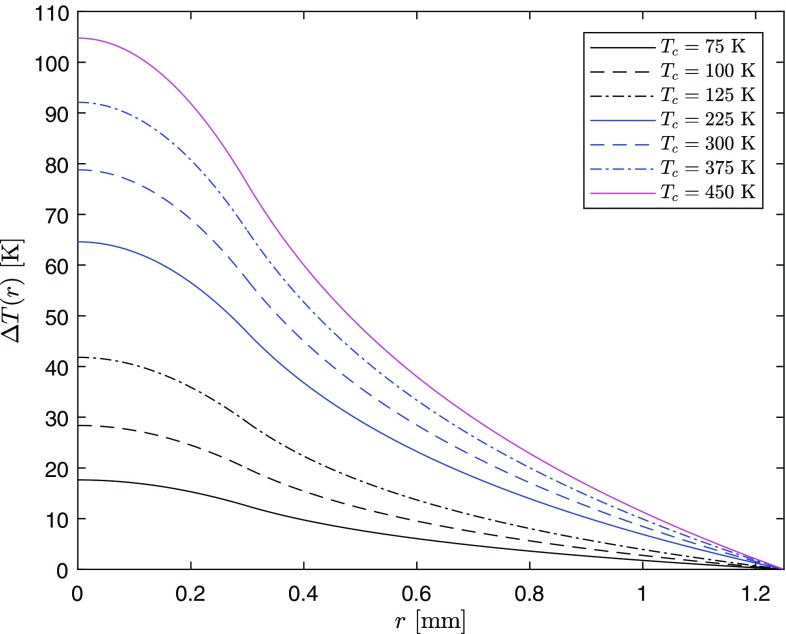
Temperature difference profile for the same thermal load conditions as Fig. 5 but with different coolant temperatures. At $T_c=225$ K, the thermal conductivity is extrapolated from the RT expression

Maintaining the same thermal load conditions, whilst lowering the temperature of the heat sink surrounding the Nd:YAG crystal, which is an artificial example as the spectroscopic proprieties of the gain medium also change [13], a decrease in the maximum temperature rise at the center of the crystal is obtained. This is due to the corresponding increase in the thermal conductivity as shown in Fig. 1. Equations (12) and (13) can be used to have a quantitative measure of this effect. In Fig. 6, the temperature rise is calculated with the same parameters above and different coolant temperatures.

### Gaussian pumping

A more realistic and often used pumping distribution employing a diffraction-limited pump is a Gaussian, which is defined as follows:

16 $$\begin{aligned} S(r,z)=Q_0e^{-2\frac{r^2}{w^2}}e^{-\alpha z}, \end{aligned}$$

where *w* is the 1/$e^2$ radius of the intensity profile of the pump beam. The volume of the heated region is given by the following:

17 $$\begin{aligned} V=\frac{\pi }{2}w^2\left( 1-e^{-2\frac{b^2}{w^2}} \right) \frac{\eta _{\mathrm{abs}}}{\alpha } \end{aligned}$$

and the term in the brackets in Eq. (17) can be set to unity in most cases of interest, where the pump beam is significantly smaller than the radius of the laser rod. Using Eqs. (9) and (16), the heat source becomes:

18 $$\begin{aligned} S(r,z)=\frac{2\eta _h P_{\text {in}}\alpha }{\pi w^2}e^{-2\frac{r^2}{w^2}}e^{-\alpha z}. \end{aligned}$$

Thus, Eq. (6) in the case of Gaussian pumping is

19 $$\begin{aligned} \frac{1}{r}\frac{\mathrm{d}U}{\mathrm{d}r}+\frac{\mathrm{d}^2U}{\mathrm{d}r^2}=-\frac{2\eta _h P_{\text {in}}\alpha }{\pi w^2}e^{-2\frac{r^2}{w^2}}e^{-\alpha z}, \end{aligned}$$

and with the following boundary conditions:

20a $$\begin{aligned}&\left. \frac{\mathrm{d}U}{\mathrm{d}r}\right| _{r=0}=0 \end{aligned}$$

20b $$\begin{aligned}&\left. \frac{\mathrm{d}U}{\mathrm{d}r}\right| _{r=b}=h\left[ T_c-T(r=b)\right] \end{aligned}$$

yields:

21 $$\begin{aligned} T(r,z)&= \left\{\vphantom{+\left[ T_c+\frac{\eta _h P_{\text {in}}\alpha e^{-\alpha z}}{2\pi bh}\right]} F_0e^{-\alpha z}\left[ \ln \left( \frac{b^2}{r^2} \right) +E_1\left( \frac{2b^2}{w^2} \right) \right. \right. \nonumber \\ & \quad \left. \left. -\;E_1\left( \frac{2r^2}{w^2} \right) \right] +\left[ T_c+\frac{\eta _h P_{\text {in}}\alpha e^{-\alpha z}}{2\pi bh}\right] ^{m+1} \right\} ^{\frac{1}{m+1}}, \end{aligned}$$

where $E_1(u)$ is the exponential integral defined as follows:

22 $$\begin{aligned} E_1(u)=\int _1^{\infty }\frac{e^{-ut}}{t}\mathrm{d}t. \end{aligned}$$

Note that if a temperature-independent thermal conductivity (i.e., $m=0$) is considered, Eq. (21) becomes

23 $$\begin{aligned} T(r,z)= & {} \frac{\eta _h P_{\text {in}}\alpha e^{-\alpha z}}{4\pi k_0}\left[ \ln \left( \frac{b^2}{r^2} \right) +E_1\left( \frac{2b^2}{w^2} \right) \right. \nonumber \\&\left. -\;E_1\left( \frac{2r^2}{w^2} \right) \right] +T_c+\frac{\eta _{h}P_{\text {in}}\alpha e^{-\alpha z}}{2\pi bh}, \end{aligned}$$

which gives a temperature difference between the center and the edge of the rod equal to

24 $$\begin{aligned} \Delta T(r,z)=\frac{\eta _h P_{\text {in}}\alpha e^{-\alpha z}}{4\pi k_0}\left[ \ln \left( \frac{b^2}{r^2} \right) +E_1\left( \frac{2b^2}{w^2} \right) -E_1\left( \frac{2r^2}{w^2} \right) \right] . \end{aligned}$$

**Fig. 7:**
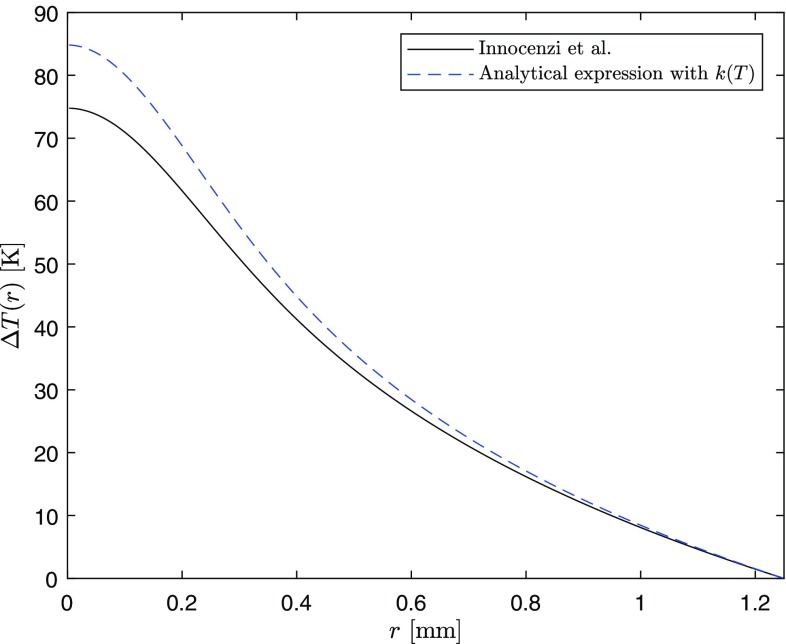
Comparison between our analytical temperature difference profile in the case of Gaussian pumping and the one published by Innocenzi [14]

For $P_h=\eta _h P_{\text {in}}$, Eq. (24) is the same result as the one published by Innocenzi et al. [14]. Figure 7 shows a comparison between our temperature profile and the one published by Innocenzi et al. with the same parameters used for the top-hat case ($w=300$ $\upmu$m is chosen). In addition, in this case, the constant thermal conductivity is evaluated using Eq. (1) at $T=T_c$.

**Fig. 8:**
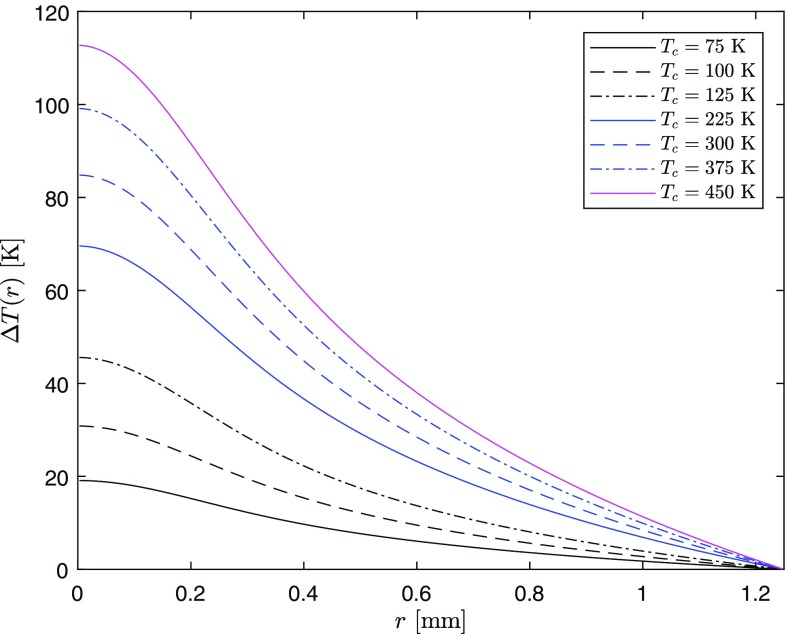
Temperature difference profile for the same thermal load conditions as Fig. 7 but with different coolant temperatures

In Fig. 8, the analytical temperature difference profiles with the same parameters above, but for different coolant temperatures, are again shown.

### Generalized *n*th order super-Gaussian

Often in the case of higher power solid-state lasers, fibre-coupled diode-laser pumps are employed. For a substantial part of the pump distribution in the laser rod, the pump can be approximated by a super-Gaussian. The heat source considered is as follows:

25 $$\begin{aligned} S(r,z)=Q_0e^{-\alpha z}e^{-2\frac{r^n}{w^n}}, \end{aligned}$$

where *n* is an even integer. The volume of the heated region can be found as follows:

26 $$\begin{aligned} V&= \int _0^{2\pi }\mathrm{d}\varphi \int _0^bre^{-2\frac{r^n}{w^n}}\mathrm{d}r\int _0^Le^{-\alpha z}\mathrm{d}z \nonumber \\&= 2\pi \frac{4^{-\frac{1}{n}}w^2}{n}\left[ \Gamma \left( \frac{2}{n},0\right) -\Gamma \left( \frac{2}{n},2\frac{b^n}{w^n}\right) \right] \frac{\eta _{\text {abs}}}{\alpha }, \end{aligned}$$

where $\Gamma (a,x)$ is the incomplete gamma function. For $n=2$, $b=1.25$ mm and $w=300$ $\upmu$m, $\Gamma \left( \frac{2}{n},2\frac{b^n}{w^n}\right) \approx 10^{-15}$, and since for higher values of *n*, this term is even smaller, it can be safely ignored. Furthermore, by definition, $\Gamma \left( \frac{2}{n},0\right) =\Gamma \left( \frac{2}{n}\right)$, so the normalized heat source is

27 $$\begin{aligned} S(r,z)=\frac{n\eta _hP_{\text {in}}\alpha }{2\pi 4^{-\frac{1}{n}}w^2\Gamma \left( \frac{2}{n}\right) } e^{-\alpha z}e^{-2\frac{r^n}{w^n}}. \end{aligned}$$

**Fig. 9:**
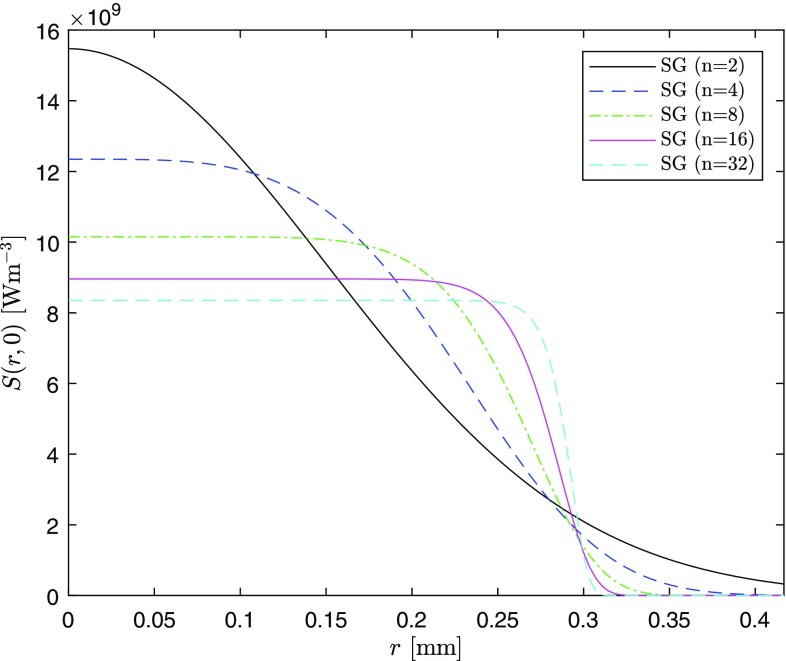
Heat source distributions in the case of super-Gaussian pumping

Figure 9 shows the heat sources for $n=2, 4, 8, 16$ and 32 at $z=0$ with the following parameter values: $w=300$ $\upmu$m, $P_{\text {in}}=25$ W, $\eta _h=0.25$, $\alpha =350$ m$^{-1}$.

Substituting Eq. (27) into Eq. (6), and using the same boundary conditions of the previous section, the temperature profile inside the end-pumped laser rod is found to be:

28 $$\begin{aligned} T(r,z)&= \left\{\vphantom{\left( T_c+\frac{\eta _h P_{\text {in}}\alpha e^{-\alpha z}}{2\pi bh}\right) ^{m+1}} G_0e^{-\alpha z}\left[ b^2{}_{2}F_{2}\left( \frac{2}{n},\frac{2}{n};1+\frac{2}{n},1+\frac{2}{n};-\frac{2b^n}{w^n}\right) \right. \right. \nonumber \\&\left. \left. -\;r^2{}_{2}F_{2}\left( \frac{2}{n},\frac{2}{n};1+\frac{2}{n},1+\frac{2}{n};-\frac{2r^n}{w^n}\right) \right] +\;\left( T_c+\frac{\eta _h P_{\text {in}}\alpha e^{-\alpha z}}{2\pi bh}\right) ^{m+1}\right\} ^{\frac{1}{m+1}},\nonumber \\ \end{aligned}$$

where

29 $$\begin{aligned} G_0=\frac{2^{\left( \frac{2}{n}-1\right) }nF_0}{\Gamma \left( \frac{2}{n}\right) w^2}, \end{aligned}$$

${}_{p}F_{q}\left( a_1,\ldots ,a_p;\,b_1,\ldots ,b_q;z\right)$ is the Generalized Hypergeometric Function, defined as

30 $$\begin{aligned} {}_{p}F_{q}\left( a_1,\ldots ,a_p;\,b_1,\ldots ,b_q;\,z\right) =\sum _{k=0}^\infty \frac{(a_1)_k \cdots (a_p)_k}{(b_1)_k \cdots (b_q)_k}\frac{z^k}{k!} \end{aligned}$$

and $(x)_k$ stands for the Pochhammer symbol, that is

31 $$\begin{aligned} (x)_k=\frac{\Gamma (x+k)}{\Gamma (x)}=x(x+1) \cdots (x+k-1) \end{aligned}$$

**Fig. 10:**
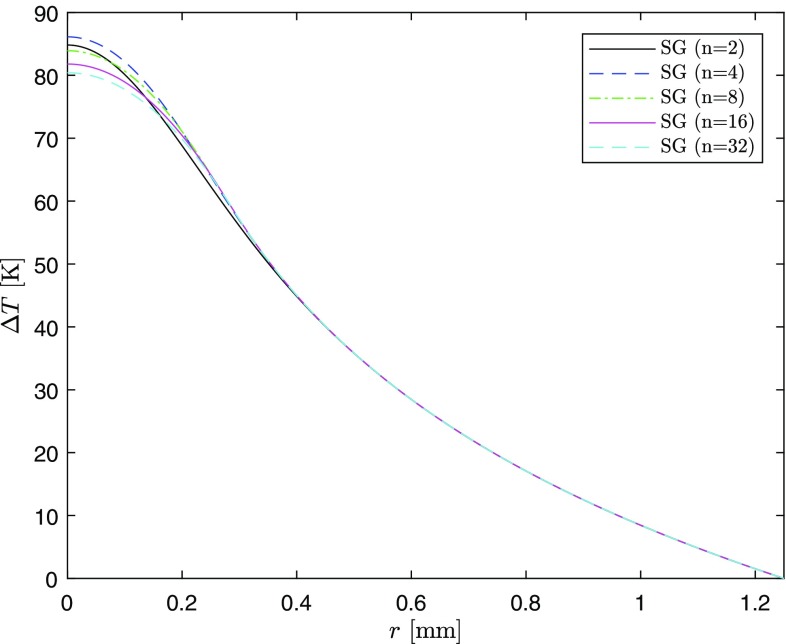
Temperature difference profile in the case of super-Gaussian pumping

**Fig. 11:**
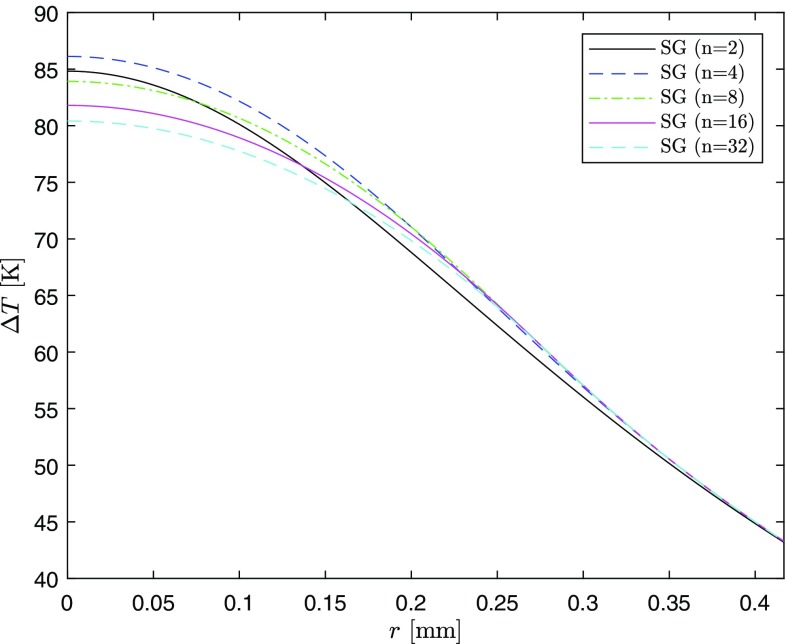
Temperature difference profile in the case of super-Gaussian pumping (zoom)

Figures 10 and 11 show Eq. (28) for $n=$2, 4, 8, 16, and 32 at $z=0$ with the same values of the parameters used in the previous sections at RT.

The center temperature is found to be maximum in the case of $n=4$. This is quite surprising, since one might have expected that it increases going from the TH ($n=\infty$) to G pump profile ($n=2$). A more in-depth analysis, varying both the transversal dimensions of the crystal and the pump laser waist in Eq. (28), shows that the relationship between the center temperatures in Figs. 10 and 11 remains the same. Indeed, this does not depend on the lab parameters, but rather on how $G_0$ and

$$\begin{aligned} {}_{2}F_{2}\left( \frac{2}{n},\frac{2}{n};1+\frac{2}{n},1+\frac{2}{n};-\frac{2b^n}{w^n}\right) \end{aligned}$$

depend on *n*.

However, *T*(0, 0) is not included in the calculation of the thermal dioptric power of the pumped rod, which does follow the expected trend (see Sect. 5). It will be shown that what matters is the coefficient of the quadratic term in the Taylor expansion of the temperature distribution, whose modulus is maximum in the case of G pumping and decreases in the SG pumping case as *n* increases.

#### Gaussian solution as the special case of the super-Gaussian with $n=2$

It can be shown for the special case of $n=2$ in Eq. (28) that its solution for the temperature profile is the same as that derived from the Gaussian distribution (Eq. 21). In this case, the Generalized Hypergeometric Function in Eq. (28) corresponds to

32 $$\begin{aligned} {}_{2}F_{2}\left( 1,1;2,2;z\right) =\sum _{k=0}^\infty \frac{(1)_k(1)_k}{(2)_k(2)_k}\frac{z^k}{k!}. \end{aligned}$$

From Eq. (31)

33a $$\begin{aligned}&(1)_k=k!\end{aligned}$$

33b $$\begin{aligned}&(2)_k=(k+1)! \end{aligned}$$

and substituting these values into Eq. (32), one obtains

34 $$\begin{aligned} {}_{2}F_{2}\left( 1,1;2,2;z\right)= & {} \sum _{k=0}^\infty \frac{1}{(k+1)^2}\frac{z^k}{k!} \nonumber \\= & {} \sum _{k=0}^\infty \frac{1}{k+1}\frac{z^{k+1}}{z(k+1)!} \nonumber \\= & {} \frac{1}{z}\sum _{k+1=1}^\infty \frac{1}{k+1}\frac{z^{k+1}}{(k+1)!}. \end{aligned}$$

By the definition of the exponential integral:

35 $$\begin{aligned} E_i(x)=\gamma +\ln |x|+\sum _{n=1}^{\infty }\frac{x^n}{nn!}, \end{aligned}$$

Equation (32) becomes:

36 $$\begin{aligned} {}_{2}F_{2}\left( 1,1;2,2;z\right) =&\frac{1}{z}\left[ -\gamma -\ln |z|+E_i(z)\right] . \end{aligned}$$

Substituting Eq. (36) into (28) with $n=2$, the temperature profile is found to be exactly as Eq. (21).

### Donut pumping

Finally, a fourth pump distribution that can be exploited to excite higher-order LG cavity modes is a donut shape [16]. This can be described by the following:

37 $$\begin{aligned} S(r,z)={\left\{ \begin{array}{ll} 0 &{} 0\le r\le d_1 \\ {\frac{\eta _h P_{\text {in}}\alpha }{\pi (d_2^2-d_1^2)}e^{-\alpha z}} &{} d_1< r\le d_2 \\ 0 &{} d_2< r\le b. \end{array}\right. } \end{aligned}$$

Using the same procedure explained in the previous sections, the following temperature distributions can be obtained, respectively, for $0\le r\le d_1$, $d_1< r\le d_2$, and $d_2< r\le b$:

38 $$\begin{aligned} T_1(z)&= \left\{ F_0e^{-\alpha z}\frac{\mathrm{d}_2^2-d_1^2+\mathrm{d}_2^2\ln \left( \frac{b^2}{\mathrm{d}_2^2} \right) -d_1^2\ln \left( \frac{b^2}{d_1^2} \right) }{d_2^2-d_1^2}\right. \nonumber \\ &\quad \left. +\;\left[ T_c+\frac{\eta _h P_{\text {in}} \alpha e^{-\alpha z}}{2\pi bh}\right] ^{m+1} \vphantom{F_0e^{-\alpha z}\frac{\mathrm{d}_2^2-d_1^2+\mathrm{d}_2^2\ln \left( \frac{b^2}{\mathrm{d}_2^2} \right) -d_1^2\ln \left( \frac{b^2}{d_1^2} \right) }{d_2^2-d_1^2}}\right\} ^{\frac{1}{m+1}} \end{aligned}$$

39 $$\begin{aligned} T_2(r,z)=\left\{ F_0e^{-\alpha z}\frac{\mathrm{d}_2^2-r^2+\mathrm{d}_2^2\ln \left( \frac{b^2}{\mathrm{d}_2^2} \right) -d_1^2\ln \left( \frac{b^2}{r^2} \right) }{d_2^2-d_1^2}+\left[ T_c+\frac{\eta _h P_{\text {in}} \alpha e^{-\alpha z}}{2\pi bh}\right] ^{m+1} \right\} ^{\frac{1}{m+1}} \end{aligned}$$

and

40 $$\begin{aligned} T_3(r,z)=\left\{ F_0e^{-\alpha z}\ln \left( \frac{b^2}{r^2}\right) +\left[ T_c+\frac{\eta _h P_{\text {in}} \alpha e^{-\alpha z}}{2\pi bh}\right] ^{m+1} \right\} ^{\frac{1}{m+1}}. \end{aligned}$$

Also, in this case, the temperature distribution reduces to the known literature-reported result if $m=0$ is imposed (see the very recent paper of Kim et al. [18]).

**Fig. 12:**
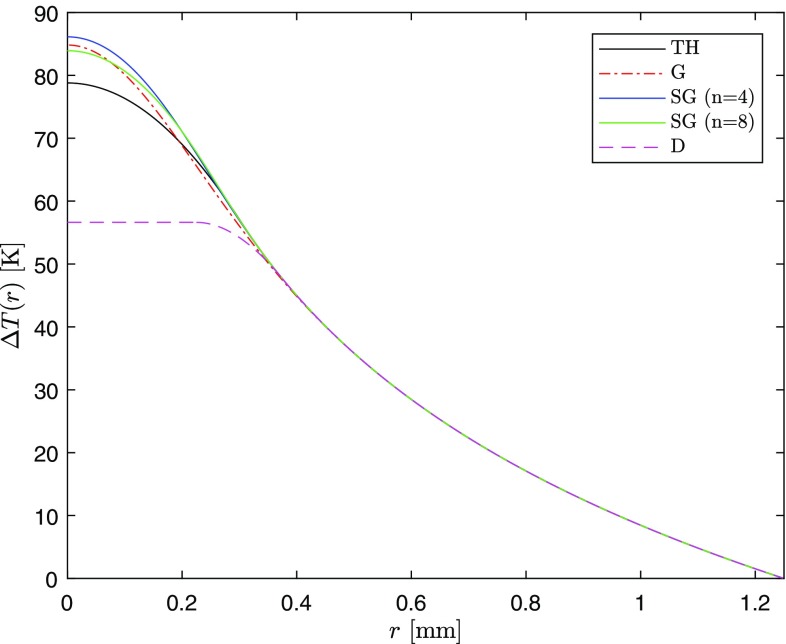
Analytical solutions of the heat equation for each distribution considered

A summary of all the analytical temperature solutions obtained at $z=0$ is shown in Fig. 12. To ensure a valid comparison with the other temperature solutions, for the donut pump, $d_1=0.225$ mm and $d_2=0.375$ mm have been chosen. In this way, the volume of the pump-photon distribution in the rod is the same for all the pump distributions.

## A brief digression on the heat transfer coefficient

The heat transfer coefficient, *h*, and its influence on the temperature distribution in this thermal model deserves a special mention. For the sake of simplicity, we limit our discussion to the pump-input surface, $z=0$, where the maximum rise of temperature is expected. In the case of a top-hat pumping distribution, from Eq. (12), the maximum temperature is given by the following:

41 $$\begin{aligned} T_1(0,0)=\left\{ F_0\left[ 1+\ln \left( \frac{b^2}{a^2} \right) \right] +\left( T_c+\frac{\eta _h P_{\text {in}}\alpha }{2\pi bh}\right) ^{m+1} \right\} ^{\frac{1}{m+1}}. \end{aligned}$$

**Fig. 13:**
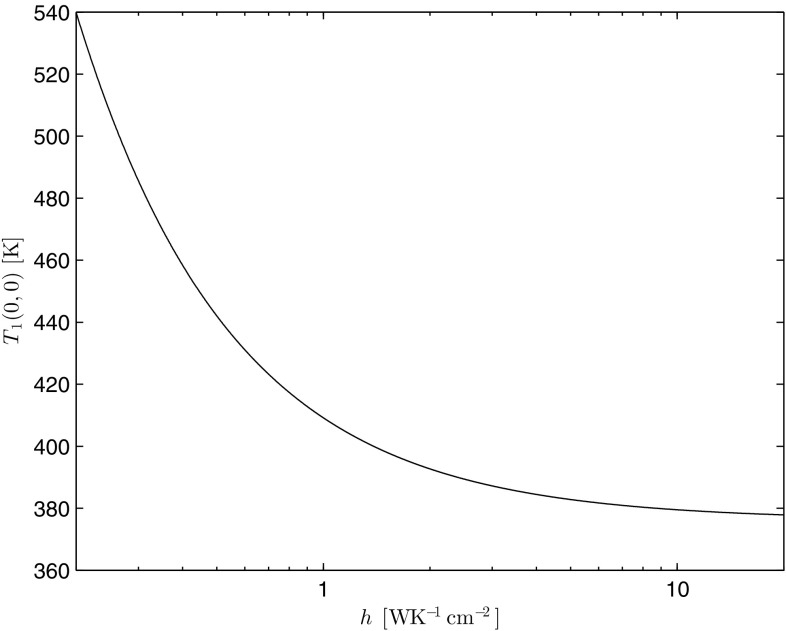
Dependence of the maximum temperature on *h*

**Fig. 14:**
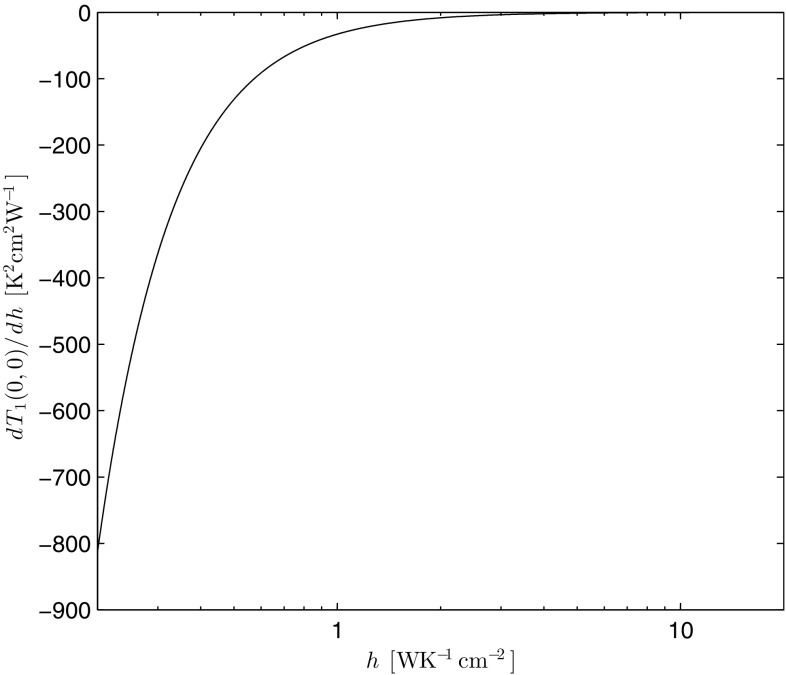
Dependence of the derivative of the maximum temperature with respect to *h* on *h*

Figures 13 and 14 show, respectively, the dependence of Eq. (41), and its derivative with respect to *h*, on *h*. Input parameters chosen are the same as in Sect. 3.1, with $T_c=300$ K.

As one can see from these figures and as one would expect, an increasing *h* implies that the maximum temperature rise asymptotes to a minimum constant value. Since this constant value is strictly reached at $h=\infty$ (i.e., in the case of perfect cooling), it is not easy to define an expression for a critical value of *h*, below which the maximum temperature rises rapidly. However, from the only *h*-dependent term in Eq. (41), one can define the limiting case, such that if

42 $$\begin{aligned} \frac{\eta _hP_{\text {in}}\alpha }{2\pi b T_c}<<h, \end{aligned}$$

then the dependence of $T_1(0,0)$ on *h* can be ignored. For the parameters used in this paper, the left-hand side of Eq. (42) is approximately equal to 0.093 WK$^{-1}$cm$^{-2}$. As it can be seen, this value is just off the scale in Figs. 13 and 14, and ultimately a tolerable increase in the peak temperature rise would have to be chosen to find a critical value for *h*. For example, a 1% increase with respect to $T_c$ (e.g. at 300 K) would imply a critical value for $h\sim 9.3$ WK$^{-1}$cm$^{-2}$. This is five times higher than the best value reported by Chénais et al. [5], when employing heat sink grease as the thermal interface to a copper mount.

### Dependence of the temperature rise on *h*

To date, the effect of *h* has only been related to the temperature difference across the boundary layer, since from the temperature-independent thermal conductivity model, the temperature difference between the center of the rod and the boundary does not depend on *h* [5]. As can be seen from Eqs. (41) and (13), this is no longer true when considering *k*(*T*), as expressed in:

43 $$\begin{aligned} T_1(0,0)-T_2(b,0)= & {} \left\{ F_0\left[ 1+\ln \left( \frac{b^2}{a^2} \right) \right] +\left( T_c+\frac{\eta _h P_{\text {in}}\alpha }{2\pi bh}\right) ^{m+1} \right\} ^{\frac{1}{m+1}}\nonumber \\&-T_c-\frac{\eta _h P_{\text {in}}\alpha }{2\pi bh} \end{aligned}$$

(note that setting $m=0$, this dependence disappears).

Actually this is quite intuitive when considering *k*(*T*), as *h*, resulting mainly in a different temperature at the boundary of the rod, therefore, changes the thermal conductivity of the rest of the laser crystal.

## Analytical expression for the thermal-lens power

Consider first the case of top-hat pumping and assume $h=\infty$, i.e., use the approximation of *perfect cooling*. A Taylor expansion can be performed at $r\approx 0$, giving

44 $$\begin{aligned} T(r,z)= & {} \left\{ F_0e^{-\alpha z}\left[ 1+\ln \left( \frac{b^2}{a^2}\right) \right] +T_c^{m+1}\right\} ^{\frac{1}{m+1}}\nonumber \\&-\;\frac{F_0e^{-\alpha z}\left\{ F_0e^{-\alpha z}\left[ 1+\ln \left( \frac{b^2}{a^2}\right) \right] +T_c^{m+1}\right\} ^{-\frac{m}{m+1}}}{a^2(m+1)}r^2 +O(r^3) \end{aligned}$$

and the approximation of $T^2(r,z)$ at $r\approx 0$ is instead

45 $$\begin{aligned} T^2(r,z)= & {} \left\{ F_0e^{-\alpha z}\left[ 1+\ln \left( \frac{b^2}{a^2}\right) \right] +T_c^{m+1}\right\} ^{\frac{2}{m+1}}\nonumber \\&-\frac{2F_0e^{-\alpha z}\left\{ F_0e^{-\alpha z}\left[ 1+\ln \left( \frac{b^2}{a^2}\right) \right] +T_c^{m+1}\right\} ^{\frac{1-m}{m+1}}}{a^2(m+1)}r^2\nonumber \\&+O(r^3). \end{aligned}$$

**Fig. 15:**
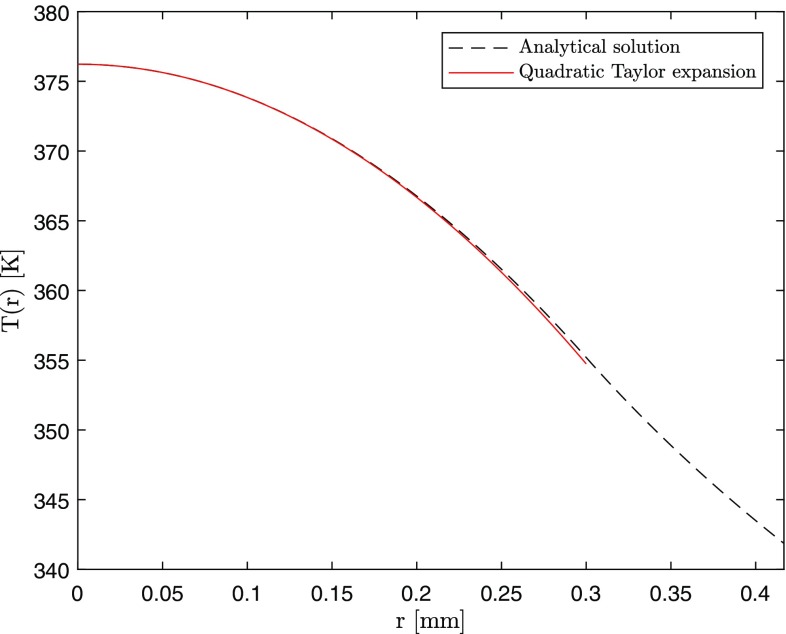
Analytical solution and its Taylor expansion inside the pumped region in the approximation of *perfect cooling* at $z=0$

Figure 15 shows the agreement of the approximate function (44) in the pumped region ($r\le a$) of the laser rod at $z=0$ for the RT.

In a similar manner as detailed by Hodgson et al. [19], we consider the thermo-optic coefficient increasing linearly with temperature, that is

46 $$\begin{aligned} \chi (T)=\chi _0+\chi _1T. \end{aligned}$$

Then, utilizing the definition for the optical path difference (OPD) [17]

47 $$\begin{aligned} \text {OPD}(r)=\int _0^L\chi (T)T(r,z)\mathrm{d}z \end{aligned}$$

and substituting Eq. (46) into Eq. (47), one obtains

48 $$\begin{aligned} \text {OPD}(r)=\chi _0\int _0^LT(r,z)\mathrm{d}z+\chi _1\int _0^LT^2(r,z)\mathrm{d}z. \end{aligned}$$

Furthermore, using

49 $$\begin{aligned} \text {OPD}(0)-\text {OPD}(r)=\frac{D_{th}r^2}{2}, \end{aligned}$$

the thermal-lens dioptric power is found to be

50 $$\begin{aligned} D_{th}= & {} \frac{2\chi _0F_0}{a^2(m+1)}\nonumber \\&\cdot \int _0^Le^{-\alpha z}\left\{ F_0e^{-\alpha z}\left[ 1+\ln \left( \frac{b^2}{a^2}\right) \right] +T_c^{m+1}\right\} ^{-\frac{m}{m+1}}\mathrm{d}z\nonumber \\&+\;\frac{4\chi _1F_0}{a^2(m+1)}\nonumber \\&\cdot \int _0^Le^{-\alpha z}\left\{ F_0e^{-\alpha z}\left[ 1+\ln \left( \frac{b^2}{a^2}\right) \right] +T_c^{m+1}\right\} ^{\frac{1-m}{m+1}}\mathrm{d}z, \end{aligned}$$

which gives (see Appendix B for details)

51 $$\begin{aligned} D_{th}= & {} \frac{2}{a^2\alpha \left[ 1+\ln \left( \frac{b^2}{a^2}\right) \right] }\nonumber \\&\cdot \left\{ \chi _0\left[ \left( F_0e^{-\alpha z}\left[ 1+\ln \left( \frac{b^2}{a^2}\right) \right] +T_c^{m+1}\right) ^{\frac{1}{m+1}}\right] _L^0\right. \nonumber \\&\left. +\;\chi _1\left[ \left( F_0e^{-\alpha z}\left[ 1+\ln \left( \frac{b^2}{a^2}\right) \right] +T_c^{m+1}\right) ^{\frac{2}{m+1}}\right] _L^0\right\} . \end{aligned}$$

Note that, thanks to the particular dependence on *r* and *z* on the temperature distribution (Eq. 44), a higher order polynomial fit equation for the thermo-optic coefficient could also be used, that is

52 $$\begin{aligned} \chi (T)=\sum _{i=0}^N\chi _iT^i, \end{aligned}$$

in which case the thermal-lens dioptric power is found to be

53 $$\begin{aligned} D_{th}= & {} \frac{2}{a^2\alpha \left[ 1+\ln \left( \frac{b^2}{a^2} \right) \right] }\sum _{i=0}^N\left\{ \chi _i\cdot \left[ (F_0e^{-\alpha z}\left[ 1+\ln \left( \frac{b^2}{a^2}\right) \right] +T_c^{m+1})^{\frac{i+1}{m+1}}\right] _L^0\right\} . \end{aligned}$$

Finally, in the case of temperature-independent thermal conductivity ($m=0$), and, thermo-optic coefficient ($\chi _1=0$), it is remarkable that Eq. (51) reduces to the well-known result for the thermal-lens dioptric power generated in a TH end-pumped laser rod [5]:

54 $$\begin{aligned} D^{\prime }_{th}=\frac{\chi _0\eta _h P_{\text {in}}\eta _{\mathrm{abs}}}{2\pi k_0. a^2} \end{aligned}$$

A similar analysis can also be followed in the case of SG pumping (and then of G if $n=2$ is considered), whereby the thermal-lens dioptric power is given by the following:

55 $$\begin{aligned} D_{th}&= \frac{2}{\alpha b^2{}_{2}F_{2}}\left\{ \chi _0\left[ \left( G_0b^2{}_{2}F_{2}e^{-\alpha z}+T_c^{m+1}\right) ^{\frac{1}{m+1}}\right] _L^0\right. \nonumber \\& \quad\left. +\;\chi _1\left[ \left( G_0b^2{}_{2}F_{2}e^{-\alpha z}+T_c^{m+1}\right) ^{\frac{2}{m+1}}\right] _L^0\right\} , \end{aligned}$$

where, for the sake of simplicity, the constant

$$\begin{aligned} {}_{2}F_{2}\left( \frac{2}{n},\frac{2}{n};1+\frac{2}{n},1 +\frac{2}{n};-\frac{2b^n}{w^n}\right) \end{aligned}$$

is indicated just with ${}_{2}F_{2}$.

Again, this result contracts to the well-known one for the thermal-lens dioptric power generated by a Gaussian-beam end-pumped laser rod in the case of constant thermal conductivity and thermo-optic coefficient ($n=2$, $m=0$ and $\chi _1=0$)[14]:

56 $$\begin{aligned} D^{\prime }_{th}=\frac{\chi _0\eta _h P_{\text {in}}\eta _{\mathrm{abs}}}{\pi k_0 w^2}. \end{aligned}$$

**Fig. 16:**
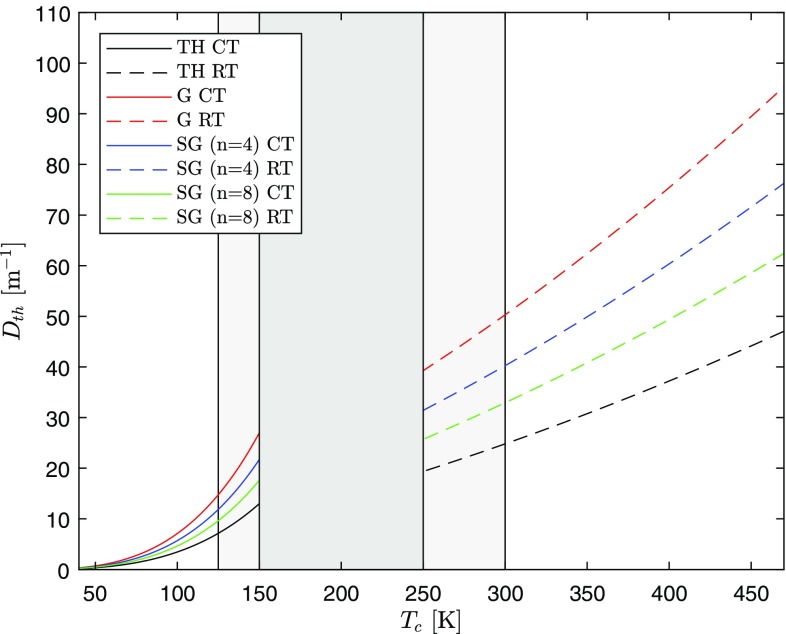
Thermal-lens dioptric power in CT and RT ranges for Top-hat, Gaussian, and super-Gaussian pumping distributions

Figure 16 shows the thermal-lens powers as a function of the coolant temperature in both ranges (CT and RT), defined in Sect. 2, for four different pump distributions with the same parameters of Fig. 4. Two different linear expressions for the thermo-optic coefficient are used, for the CT and RT ranges, corresponding to the valid temperature ranges for the thermal conductivity fits. The values utilized for $\chi _0$ and $\chi _1$ for each range have been found as follows.

Consider the thermo-optic coefficient defined as follows [17]:

57 $$\begin{aligned} \chi (T)=\frac{\mathrm{d}n}{\mathrm{d}T}(T)+C_{\alpha }(n_0-1)(1+\nu )\alpha _T(T), \end{aligned}$$

where $\mathrm{d}n/\mathrm{d}T(T)$ is the thermal dispersion, $C_{\alpha }$ is a parameter between 0 and 1 that includes the limitation of a heated element of the rod in the free expansion along the longitudinal direction, due to the colder surrounding, if a transversely localized temperature increase occurs, $\nu$ is the Poisson’s ratio, $n_0$ is the refractive index, and $\alpha _T(T)$ the thermal expansion coefficient. Supposing both the thermal dispersion and the thermal expansion coefficient linearly depend upon temperature, that is

58 $$\begin{aligned} \frac{\mathrm{d}n}{\mathrm{d}T}(T)=l_0+l_1T \end{aligned}$$

and

59 $$\begin{aligned} \alpha _T(T)=l_2+l_3T. \end{aligned}$$

Equation (57) becomes

60 $$\begin{aligned} \chi (T)&= l_0+C_{\alpha }(n_0-1)(\nu +1)l_2\nonumber \\&\quad+\;[l_1+C_{\alpha }(n_0-1)(\nu +1)l_3]T, \end{aligned}$$

that is, equivalent to Eq. 46, by defining

61 $$\begin{aligned} \chi _0=l_0+C_{\alpha }(n_0-1)(\nu +1)l_2 \end{aligned}$$

and

62 $$\begin{aligned} \chi _1=l_1+C_{\alpha }(n_0-1)(\nu +1)l_3. \end{aligned}$$

For the sake of simplicity, $C_{\alpha }$ is set equal to 1 [17], and, since for YAG $\nu =0.25$ and $n_0\sim 1.8$, Eqs. (61) and (62), therefore, become

63 $$\begin{aligned} \chi _0=l_0+l_2 \end{aligned}$$

and

64 $$\begin{aligned} \chi _1=l_1+l_3. \end{aligned}$$

$l_0$, $l_1$, $l_2$ and $l_3$ are obtained from a best fit of Eqs. (58) and (59) to measured data, as reported by Furuse et al. [20] for a single-crystal YAG (except the $\mathrm{d}n/\mathrm{d}T$ coefficient at RT, the data of which were for ceramic YAG). Their values are listed in Table 2.

**Table 2:** Fit parameters for the linear model of the thermo-optic coefficient

Range	$l_0\left[ \frac{10^{-6}}{\text {K}}\right]$	$l_1\left[ \frac{10^{-8}}{\text {K}^2}\right]$	$l_2\left[ \frac{10^{-6}}{\text {K}}\right]$	$l_3\left[ \frac{10^{-8}}{\text {K}^2}\right]$
CT	−2.3$^\mathrm{a}$	3.8$^\mathrm{a}$	0.18$^\mathrm{b}$	2.1$^\mathrm{b}$
RT	3.4$^\mathrm{c}$	1.7$^\mathrm{c}$	4.2$^\mathrm{d}$	0.62$^\mathrm{d}$

References: $$^\mathrm{a}$$ [21], $$^\mathrm{b}$$ [6], $$^\mathrm{c}$$ [20] and $$^\mathrm{d}$$ [22]

Note that the $\mathrm{d}n/\mathrm{d}T$ data were obtained using a 633 nm He–Ne laser [20]; however, for a real understanding of the lens power, it would need to be evaluated with data measured at the laser wavelength. As reported by Sato and Taira [22] at RT, for example, at a wavelength of 1064 nm, the $\mathrm{d}n/\mathrm{d}T$ value is 12.1 $10^{-6}/\text {K}$, ${\sim }50\%$ higher than the value at 633 nm.

The lens power in Fig. 16 at a heatsink temperature $T_c>125$ K in the CT range should be taken with caution, since, in this case, the maximum temperature of the rod no longer belongs to the CT range and the fit parameters $T_0$, $k_0$ and $m_{\text {CT}}$ are not strictly valid anymore (see Figs. 6 and 8).

## Conclusion

In this paper, we have drawn on the fact that the thermal conductivity of laser gain media is dependent upon their temperature. Consequently, solving the heat conductance equation, using the Kirchhoff integral transform, analytical expressions were derived for a number of realistic pumping sources, typically used in end-pumped rod-laser configurations. We compare the solutions obtained with exemplars from the literature and show that in the limit of a constant thermal conductivity, there is a natural convergence.

Through the power of having an analytical solution, we show that the temperature rise at the center of the laser rod has a dependence on the thermal impedance of the boundary layer, as is intuitively expected. In contrast with accepted expressions, these solutions predict an additional temperature rise associated with the thermal conductance parameter *h*. With this expression, guidance on the minimum acceptable value for *h* for a set of design parameters can be obtained.

Finally, analytical expressions for the thermal-lens dioptric power are derived in the limit of perfect boundary conditions. Intimately connected with the temperature dependence of the thermal conductivity, in the limit of a constant $k_0$, the expressions once again converge to their simplified analogues for Gaussian or top-hat pumping distributions.

It is evident that the solutions derived herein, for the typical parameters chosen, demonstrate a higher temperature rise than those obtained with temperature-independent thermal conductivity reported previously. Having analytical expressions that can be used for a range of temperatures provide a powerful set of tools for designing future end-pumped laser systems. This will be especially relevant to laser systems operating at cryogenic temperatures.

## Appendices

### Appendix A: Calculation of the temperature distribution in a top-hat end-pumped laser rod

In the case of top-hat pumping and considering the inner region of the laser rod, $0\le r\le a$, Eq. (6) becomes

65 $$\begin{aligned} \frac{1}{r}\frac{\mathrm{d}U_1(r,z)}{\mathrm{d}r}+\frac{\mathrm{d}^2U_1(r,z)}{\mathrm{d}r^2}=-\;Q_0e^{-\alpha z}, \end{aligned}$$

where the subscript 1 indicates that we are focusing just on the inner region.

Let $\frac{\mathrm{d}U_1(r,z)}{\mathrm{d}r}=\nu _1(r)$, which gives $\frac{\mathrm{d}^2U_1(r,z)}{\mathrm{d}r^2}=\frac{\mathrm{d}\nu _1(r)}{\mathrm{d}r}$. Multiply both sides by *r* and substitute $1=\frac{\mathrm{d}r}{\mathrm{d}r}$, giving

66 $$\begin{aligned} \frac{\mathrm{d}r}{\mathrm{d}r}\nu _1(r)+r\frac{\mathrm{d}\nu _1(r)}{\mathrm{d}r}=-\;Q_0e^{-\alpha z}r. \end{aligned}$$

Therefore, Eq. (66) corresponds to

67 $$\begin{aligned} \frac{\mathrm{d}}{\mathrm{d}r}[r\nu _1(r)]=-\;Q_0e^{-\alpha z}r \end{aligned}$$

and performing an integration in both sides, one obtains

68 $$\begin{aligned} r\nu _1(r)=-\;\frac{Q_0e^{-\alpha z}}{2}r^2+A, \end{aligned}$$

where *A* is a constant of integration, which must be found with a boundary condition. Dividing both sides by *r*, Eq. (68) becomes

69 $$\begin{aligned} \nu _1(r)=\frac{\mathrm{d}U_1(r,z)}{\mathrm{d}r}=-\;\frac{Q_0e^{-\alpha z}}{2}r+\frac{A}{r} \end{aligned}$$

and, with another integration, it returns

70 $$\begin{aligned} U_1(r,z)=-\;\frac{Q_0e^{-\alpha z}}{4}r^2+A\ln (r)+B, \end{aligned}$$

where *B* is another constant of integration. By the definition of *U*(*r*), Eq. (7)^1^

71 $$\begin{aligned} \frac{k_0}{T_0^m(m+1)}T_1^{m+1}(r,z)=-\;\frac{Q_0e^{-\alpha , z}}{4}r^2+A\ln (r)+B \end{aligned}$$

which gives

72 $$\begin{aligned} T_1(r,z)=\left\{ \left[ -\frac{Q_0e^{-\alpha z}}{4}r^2+A\ln (r)+B\right] \frac{T_0^m(m+1)}{k_0}\right\} ^{\frac{1}{m+1}}. \end{aligned}$$

For the outer region of the rod, $a< r\le b$, Eq. (6) becomes

73 $$\begin{aligned} \frac{1}{r}\frac{\mathrm{d}U_2(r,z)}{\mathrm{d}r}+\frac{\mathrm{d}^2U_2(r,z)}{\mathrm{d}r^2}=0 \end{aligned}$$

and it gives a solution of the form

74 $$\begin{aligned} U_2(r,z)=D\ln (r)+E \end{aligned}$$

and then

75 $$\begin{aligned} T_2(r,z)=\left\{ \left[ D\ln (r)+E\right] \frac{T_0^m(m+1)}{k_0}\right\} ^{\frac{1}{m+1}}. \end{aligned}$$

Now, the boundary equations must be used to find the constants of integration *A*, *B*, *D*, and *E*.

For reasons of symmetry, the amount of heat conducted per unit area (heat flux) in the *r* direction must be equal to 0 at the rod center, that is

76 $$\begin{aligned} -\;k(T)\left. \frac{\mathrm{d}T_1}{\mathrm{d}r}\right| _{r=0}=0. \end{aligned}$$

Substituting Eq. (5) into Eq. (76) and using the chain rule of differentiation, the first boundary condition (76) becomes:

77 $$\begin{aligned} -\left. \frac{\mathrm{d}U_1}{\mathrm{d}r}\right| _{r=0}=0 \end{aligned}$$

and, by Eq. (69), it gives

78 $$\begin{aligned} A=0. \end{aligned}$$

At the external surface of the rod, $r=b$, the boundary condition is chosen instead to be [15]:

79 $$\begin{aligned} -\left. \frac{\mathrm{d}U_2}{\mathrm{d}r}\right| _{r=b}=h\left[ T_2(r=b)-T_c\right] \end{aligned}$$

Substituting in Eqs. (74) and (75), it returns

80 $$\begin{aligned} \frac{D}{bh}=T_c-\left\{ \left[ D\ln (b)+E\right] \frac{T_0^m(m+1)}{k_0}\right\} ^{\frac{1}{m+1}}. \end{aligned}$$

Finally, the continuity of the temperature and its derivative at $r=a$ is imposed, that is

81 $$\begin{aligned} T_1(r=a)=T_2(r=a), \end{aligned}$$

which gives

82 $$\begin{aligned} -\frac{Q_0e^{-\alpha z}}{4}a^2+B=D\ln (a)+E \end{aligned}$$

and

83 $$\begin{aligned} \left. \frac{\mathrm{d}T_1}{\mathrm{d}r}\right| _{r=a}=\left. \frac{\mathrm{d}T_2}{\mathrm{d}r}\right| _{r=a}, \end{aligned}$$

which gives

84 $$\begin{aligned}&-\frac{Q_0e^{-\alpha z}a}{2}\left[ \frac{T_0^m(m+1)}{k_0}\left( -\frac{Q_0e^{-\alpha z}a^2}{4}+B\right) \right] ^{-\frac{m}{m+1}}\nonumber \\&\quad =\frac{D}{a}\left[ \frac{T_0^m(m+1)}{k_0}\left( D\ln (a) +E\right) \right] ^{-\frac{m}{m+1}}. \end{aligned}$$

Equations (80), (82) and (84) can be solved to find *B*, *D*, and *E*, and substituting their values in Eqs. (72) and (75), one obtains

85 $$\begin{aligned} T_1(r,z)= & \left\{\vphantom{+\;\left( T_c+\frac{\eta _h P_{\text {in}} \alpha e^{-\alpha z}}{2\pi bh} \right) ^{m+1}}F_0e^{-\alpha z}\left[ 1-\frac{r^2}{a^2}+\ln \left( \frac{b^2}{a^2} \right) \right] \right. \nonumber \\ & \left. +\;\left( T_c+\frac{\eta _h P_{\text {in}} \alpha e^{-\alpha z}}{2\pi bh} \right) ^{m+1} \right\} ^{\frac{1}{m+1}} \end{aligned}$$

86 $$\begin{aligned} T_2(r,z)= & \left[\vphantom{+\;\left( T_c+\frac{\eta _h P_{\text {in}} \alpha e^{-\alpha z}}{2\pi bh}\right) ^{m+1} } F_0e^{-\alpha z}\ln \left( \frac{b^2}{r^2} \right) \right. \nonumber \\ & \left. +\;\left( T_c+\frac{\eta _h P_{\text {in}} \alpha e^{-\alpha z}}{2\pi bh}\right) ^{m+1} \right] ^{\frac{1}{m+1}}. \end{aligned}$$

### Appendix B: Analytical calculation of the thermal-lens dioptric power in the case of top-hat pumping

The integrals in Eq. (50) can be analytically solved with two changes of variable. Here, only the steps used to solve the first one are shown, since the other integral can be solved in exactly the same way.

We, therefore, consider

87 $$\begin{aligned} I_1&= \frac{2\chi _0F_0}{a^2(m+1)}\nonumber \\&\cdot \int _0^Le^{-\alpha z}\left\{ F_0\left[ 1+\ln \left( \frac{b^2}{a^2}\right) \right] e^{-\alpha z}+T_c^{m+1}\right\} ^{-\frac{m}{m+1}}\mathrm{d}z. \end{aligned}$$

Introducing $$u=-\alpha z$$ ($$\mathrm{d}u=-\alpha \mathrm{d}z$$)

88 $$\begin{aligned} I_1= & {} -\frac{2\chi _0 F_0}{a^2\alpha (m+1)}\nonumber \\&\cdot \int _0^L e^u\left( F_0\left[ 1+\ln \left( \frac{b^2}{a^2}\right) \right] e^u+T_c^{m+1}\right) ^{-\frac{m}{m+1}}\mathrm{d}u \end{aligned}$$

and using a new change of variable

$$\begin{aligned} w=F_0\left[ 1+\ln \left( \frac{b^2}{a^2}\right) \right] e^u+T_c^{m+1} \end{aligned}$$

$$\left( \mathrm{d}w=F_0\left[ 1+\ln \left( \frac{b^2}{a^2}\right) \right] e^udu\right)$$, one obtains

89 $$\begin{aligned} I_1= & {} -\frac{2\chi _0 }{a^2(m+1)\alpha \left[ 1+\ln \left( \frac{b^2}{a^2}\right) \right] }\nonumber \\&\cdot \int _0^Lw^{-\frac{m}{m+1}}\mathrm{d}w\nonumber \\&=-\frac{2\chi _0 }{a^2\alpha \left[ 1+\ln \left( \frac{b^2}{a^2}\right) \right] } \left[ w^{\frac{1}{m+1}}\right] _0^L, \end{aligned}$$

so

90 $$\begin{aligned} I_1= & {} \frac{2\chi _0}{a^2\alpha \left[ 1+\ln \left( \frac{b^2}{a^2}\right) \right] }\nonumber \\&\cdot \left[ \left( F_0e^{-\alpha z}\left[ 1+\ln \left( \frac{b^2}{a^2}\right) \right] +T_c^{m+1} \right) ^{\frac{1}{m+1}}\right] _L^0. \end{aligned}$$

## References

[1] Giesen A, Speiser J (2007). IEEE J. Sel. Top. Quantum Electron..

[2] Richardson DJ, Nilsson J, Clarkson WA (2010). J. Opt. Soc. Am. B Opt. Phys..

[3] Rutherford TS, Tulloch WM, Gustafson EK, Byer RL (2000). IEEE J. Quantum Electron..

[4] Clarkson WA (2001). J. Phys. D Appl. Phys..

[5] Chénais S, Duron F, Forget S, Balembois F, Georges P (2006). Prog. Quantum Electron..

[6] Aggarwal RL, Ripin DJ, Ochoa JR, Fan TY (2005). J. Appl. Phys..

[7] Moghtader Dindarlu MH, Maleki A, Saghafifar H, Kavosh Tehrani M, Baghali S (2015). Laser Phys..

[8] Bagnall KR, Muzychka YS, Wang EN (2014). Trans. Componen. Packag. Manuf. Technol..

[9] Hodgson N, Weber H (1993). IEEE J. Quantum Electron..

[10] L. Cini, W.O.S. Bailey, Y. Yang, J.I. Mackenzie, temperature-dependent analytical thermal model for end-pumped solid-state lasers. JM5A.5 at Advanced Solid State Lasers (ASSL), OSA (2017) Nagoya, Japan 10.1007/s00340-017-6848-y PMC6956956 31997852

[11] Sato Y, Akiyama J, Taira T (2008). Opt. Mater..

[12] Fan TY, Ripin DJ, Aggarwal RL, Ochoa JR, Chann B, Tilleman M, Spitzberg J (2007). IEEE J. Sel. Top. Quantum Electron..

[13] Yoon SJ, Mackenzie JI (2014). Opt. Express.

[14] Innocenzi ME, Yura HT, Fincher CL, Fields RA (1990). Appl. Phys. Lett..

[15] Cousins A (1992). IEEE J. Quantum Electron..

[16] Kim JW, Mackenzie JI, Hayes JR, Clarkson WA (2011). Opt. Express.

[17] Bjurshagen S, Koch R (2004). Appl. Opt..

[18] Kim DJ, Noh SH, Ahn SM, Kim JW (2017). Opt. Express.

[19] Hodgson N, Weber H (2005). Laser Resonators and Beam Propagation, Chapter 13.2.6.

[20] Furuse H, Yasuhara R, Hiraga K (2014). Opt. Mater. Express.

[21] Wynne R, Daneu JL, Fan TY (1999). Appl. Opt..

[22] Sato Y, Taira T (2014). Opt. Mater. Express.

